# Using implementation mapping to optimize the impact of Universal School meals: a type III hybrid implementation-effectiveness study protocol

**DOI:** 10.1186/s43058-025-00769-y

**Published:** 2025-10-01

**Authors:** Gabriella M. McLoughlin, Angel Smith, Alex R. Dopp, Resa Jones, Omar Martinez, Shiriki Kumanyika, Recai Yucel, Ross C. Brownson, Jennifer Orlet Fisher

**Affiliations:** 1 Department of Social and Behavioral Sciences, Temple University College of Public Health, Philadelphia, PA USA; 2 RAND, Santa Monica, CA USA; 3 Department of Epidemiology and Biostatistics, Temple University College of Public Health, Philadelphia, PA USA; 4 Fox Chase Cancer Center, Temple University Health, Philadelphia, PA USA; 5 College of Medicine, University of Central Florida, Orlando, FL USA; 6 Perelman School of Medicine, University of Pennsylvania, Philadelphia, PA USA; 7 Siteman Cancer Center, Washington University School of Medicine, St. Louis, MO USA; 8 School of Public Health, Washington University in St. Louis, St. Louis, MO USA

**Keywords:** Universal school meals, Food insecurity, Obesity policy, Equity, Implementation mapping, Implementation strategies, Community-engaged research

## Abstract

**Background:**

Provision of government subsidized school meals at no charge to all students in income-eligible schools (Universal School Meals) is a critical policy approach to address food insecurity and risk for obesity in school-aged children. However, despite documented benefits, implementation challenges remain, which limit the uptake and associated impact of this provision. To ensure the longevity of this policy approach, equity-focused solutions that center the needs of those tasked with implementation and the most vulnerable Universal School Meals recipients are necessary. The aims of this study are to develop equity-focused implementation strategies and test them through a hybrid type III cluster-randomized trial to examine potential effectiveness on improving student uptake and implementation across the school system.

**Methods:**

Aim 1 will comprise the first tasks of Implementation Mapping to co-develop implementation strategies in partnership with school implementers and recipients to ensure contextual fit within their school system. Aim 2 will comprise the final step of implementation mapping with a hybrid type III implementation-effectiveness trial to examine primary implementation and effectiveness outcomes of the applied strategies. Reach and penetration will be the primary implementation outcomes in addition to acceptability, feasibility, cost, and sustainability. Health outcomes comprise family food security, student dietary behaviors, and body mass index. Baseline, 6-month, and 12-month assessments will be recorded. A convergent (Quantitative–Qualitative) mixed methods design will be employed for analysis; exploratory hierarchical multiple regression models will be run for each behavioral outcome using students as the unit of observation and schools as the unit of analysis. Survey and interview data for implementation outcomes will be analyzed deductively according to the Exploration, Preparation, Implementation, and Sustainment and Getting to Equity frameworks then inductively to generate overarching themes across the trial period.

**Discussion:**

This implementation mapping process will yield equity-driven strategies, which can be successfully implemented in school settings to improve uptake of USM and reduce food insecurity and obesity-related disparities in high-risk youth. This study presents a rigorous and equity-driven implementation research agenda with the potential to advance school-based obesity prevention efforts by identifying, developing, and evaluating context-specific strategies that meet the needs of vulnerable student populations.

**Trial registration:**

ClinicalTrials.gov, NCT06579079, Registered on 11–5-2024.

**Supplementary Information:**

The online version contains supplementary material available at 10.1186/s43058-025-00769-y.

Contributions to the literature

- This study marks a much-needed alignment of implementation science and health equity to address disparities in food insecurity and obesity risk through community-engaged implementation mapping methodology
- Partnerships created with a local school district facilitate the use of natural experiment research so that we can study the impact of the Universal School Meals implementation strategy in real-time and commence efforts to scale up strategies to be applied across the district starting immediately after the trial.
- This study uniquely contributes to the literature by examining Universal School Meals implementation in an urban city with large racial and ethnic minority populations, providing urgently needed evidence on how to tailor and sustain school-based nutrition interventions in historically marginalized communities most affected by structural inequities in food access and health outcomes.

## Background

Children living in low-income situations are more than twice as likely to experience food insecurity than more affluent counterparts [1–3]. Risk for food insecurity is heavily linked to risk for obesity; [4] this relationship is heightened in populations with low income. Given the complex, community and population-level factors that influence health outcomes (i.e., poverty, discrimination, inadequate access to healthy food) [5], policy, systems, and environmental (PSE) approaches are necessary to mitigate obesity risk and achieve equitable outcomes for socially and economically marginalized populations such as and racial/ethnic minorities and those with low-income [6–8]. School-based PSE interventions, such as those which promote healthy eating and physical activity through enhancing the school environment, show promise for preventing obesity [9–12]. However, this impact remains limited because most interventions have not been designed with consideration of long-term implementation and sustainability [13, 14].

For the last six decades in the US, the National School Lunch Program [15] and the National Breakfast Program [16] (NSLBP) have been combatting food insecurity among children with low-income backgrounds. These programs are the primary federal food safety net for school-aged children. Universal School Meals (USM) operate through a policy called Community Eligibility Provision, which allows all schools and districts serving more than 25% low-income students to provide free breakfast and lunch under the NSLBP [17]. Several states have now moved to state-wide USM to address growing rates of food insecurity [18]. Research has shown that providing healthy school meals to students via USM is associated with higher quality nutritional intake and reduced obesity prevalence, especially in low-income students [8, 19–21]. Thus, increasing access to healthy meals at school is a critical step to mitigating disparities in obesity prevalence in youth [20]. USM adoption is also positively associated with quality of dietary intake, food security, and academic achievement outcomes observed through randomized trials and longitudinal studies [22–24]. Therefore, USM is a key PSE approach for equitable obesity prevention.

Despite the many benefits associated with USM, schools cite logistical challenges (i.e., lack of staffing for implementation, limited space) and lack of uptake among students [25]. Reports highlight consistent increases in adoption among eligible schools and districts over the last 10 years, [17] yet student participation in USM remains low; available data indicate only 30–40% of students partake in breakfast and 50–60% in lunch [26]. These trends are reflected in the School District of Philadelphia (SDP) [27, 28]. Programs and policies designed to mitigate health disparities for food insecurity and obesity cannot make the most impact if they are not reaching their target population. Because the federal reimbursement rate for schools is tied directly to participation (i.e., the more meals taken, the more reimbursement the district receives), maintaining reach is critical to making USM financially feasible. Students who do not participate in school meals are more likely to purchase unhealthy foods from outside retailers (e.g., corner stores), [29, 30] increasing risk for overweight and obesity [31]. Negative impacts on school climate, [32] school finances, [24] and household food insecurity [24, 33] highlight the need for efforts to increase reach among low-income youth, especially adolescents where prevalence of food insecurity and obesity are highest [34]. Thus, optimizing reach of USM will enhance its impact on addressing disparities in child obesity.

Dissemination and implementation science facilitates the process by which evidence-based interventions (EBIs) are implemented and sustained in practice [35, 36]. Through this lens, the desired outcome is implementation effectiveness as a means to reach clinical effectiveness (i.e., obesity prevention). This is achieved by developing implementation strategies, which are designed to enhance implementation of EBIs [37]. Such strategies can be chosen through a variety of ways, but implementation mapping is a key method to ensure a community-driven process [38]. Implementation mapping is based on intervention mapping and instead of developing new interventions, focuses on co-creation of implementation strategies through accomplishing five key tasks: 1) Needs Assessment; 2) Identify Outcomes; 3) Select Implementation Strategies; 4) Develop Implementation Protocols; and 5) Evaluate Outcomes. Although implementation science provides systematic approaches for increasing real-world impact of obesity prevention, health equity is not explicitly considered [39]. Recent advancements have introduced health equity as a key focus [40–43], including the Consolidated Framework for Implementation Research (CFIR version 2) [44, 45] and the Getting to Equity (GTE) [7] framework for obesity prevention, which stresses that reducing disparities requires community-engaged strategies that 1) provide healthy options (e.g., healthy school meals), 2) reduce barriers (e.g., stigma), 3) improve individual social and economic resources, and 4) build on community assets and capacity [7]. Accordingly, leveraging implementation strategies to improve USM implementation is critical for equitable access to USM [46].

Given our previously conducted needs assessment [47], this study will accomplish the development and testing of an equity-focused implementation strategy for USM to promote improved uptake of healthy school meals among school students in Philadelphia, United States. Specifically, the aims of this study are to:

- Aim 1: Use a community-engaged procedure to develop an equity-focused implementation strategy through a cluster randomized trial.
- Aim 2: Evaluate implementation and student-level outcomes of the equity-focused strategy using a hybrid type III implementation-effectiveness design.

This pilot intervention will utilize an evidence-based process (i.e., implementation mapping) [38] and test primary implementation outcomes of penetration and cost, [48, 49] and secondary impact on student health outcomes (i.e., weight status, food security, dietary behaviors).

## Methods

In close partnership with the SDP this study encompasses a rigorous implementation mapping procedure to improve the implementation and public health impact of USM within SDP schools [38, 50]. This pilot project began with identifying key determinants of implementation and desired outcomes through a rigorous needs assessment which accomplished Task 1 of implementation mapping [47]. This task was completed intentionally before the trial to allow time for the research team to 1) build partnerships with schools in the district and show investment (which took considerable time) and 2) conduct a needs assessment with a larger sample of schools prior to setting up a cluster-randomized trial.

### Theoretical framework and preliminary findings

**Fig. 1:**
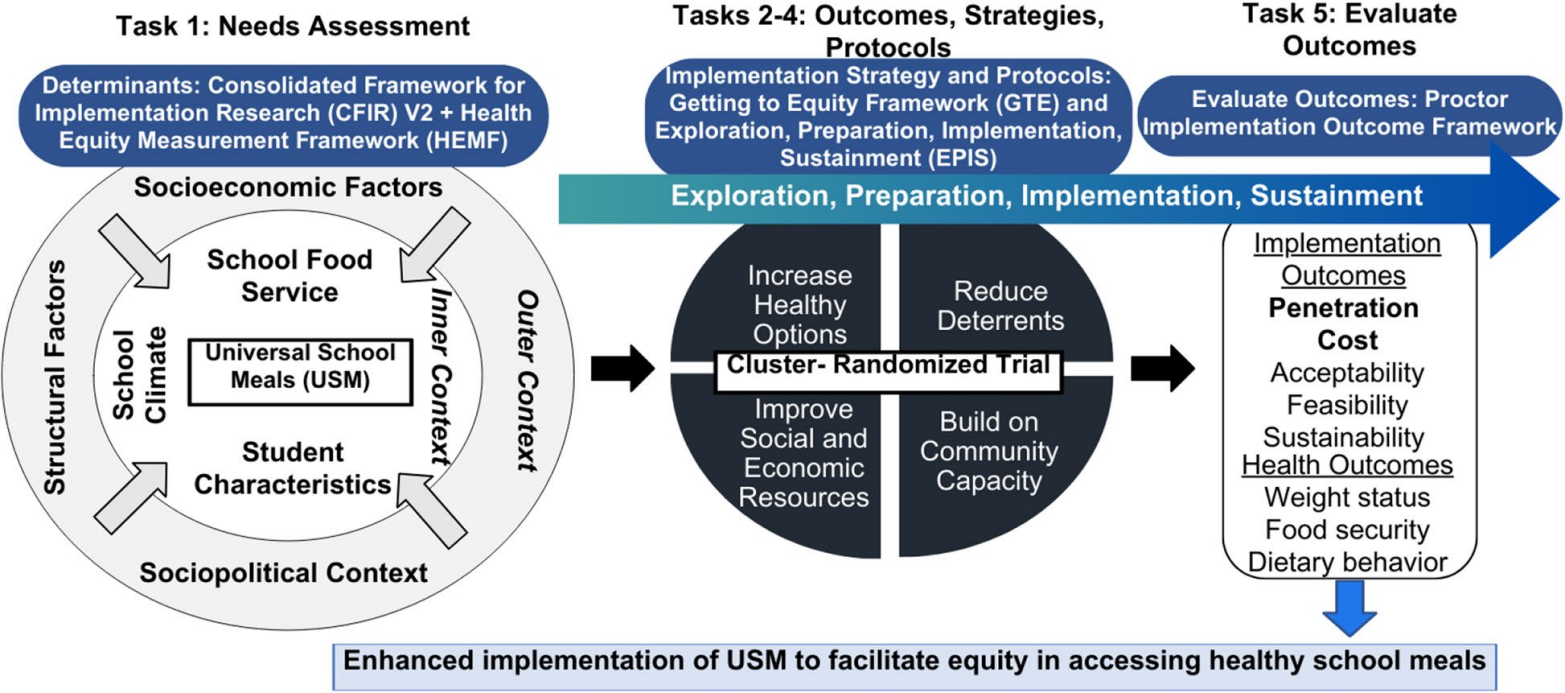
Conceptual overview of the implementation mapping project

Figure 1 depicts the conceptual framework for our implementation mapping process, which is described in full below.

Task 1 identified key determinants of USM implementation for addressing health disparities across 8 schools (6 elementary/middle; 2 high) within SDP. This task was completed over the 2023–2025 school years, grounded in the CFIR (v2) [51] and Health Equity Measurement Framework (HEMF) [52] and was recently published [47]. Among 8 schools, 193 participants completed surveys and/or interviews grounded in the CFIR including students in middle (14%) and high school (10%) grades, parents (26%), food service staff/managers (11%), teachers (25%), and administrators (14%). Findings revealed significant concerns with food insecurity expressed by participants at each level of the school system, and viewpoints among parents and staff that USM is essential for ensuring students are ready for learning. Key barriers were perceptions among students and parents that their views are not prioritized in USM implementation, which could limit desires to participate in school meals. Participants in all groups discussed challenges in uptake due to students bringing food in from competitive vendors (i.e., corner stores & fast food restaurants) and a lack of affordable grocery stores in the school vicinity, limiting students’ socialization to healthier foods such as those provided in the school menu [47]. These findings heavily inform our protocol design and methods.

The GTE [7] and the Exploration, Preparation, Implementation, Sustainment (EPIS) [53] frameworks will ground Aim 1 (Tasks 2–4) to guide the selection and tailoring of implementation strategies and protocols [7, 54]. The GTE was developed to inform operationalization of equity principles and concepts in PSE approaches related to obesity, in partnership with members of the community of interest. GTE guidance prompts for identifying and addressing intervention design features and contextual variables to improve equity impact, accounting for individual and community-level resources and capacity [7]. The EPIS framework [53] conceptualizes key phases of Exploration (deliberating ways to innovate/the innovation), Preparation (planning for implementation), Implementation (the process of implementing), and Sustainment (maintaining implementation) that guide and describe the implementation process. Similar to CFIR, EPIS provides a set of constructs among key domains of outer context (outside the school system) and inner (within school) context across these phases, bridging factors that span across inner and outset settings, innovation factors, and role of innovation adopters. Together, these frameworks will support the co-development process, ensuring that strategies and protocols developed are grounded in addressing inequities in access (GTE), and that we have a strong structure (EPIS) that facilitates consideration of key determinants and processes over discrete phases of the mapping process.

Primary implementation outcomes aligning with Proctor et al.’s framework [55] will be collected in Aim 2 (Task 5) to evaluate the resultant implementation strategy through a Hybrid Type III Design [56]. Chosen outcomes are penetration (i.e., degree to which school meals were provided equitably to low-income students) and cost (i.e., time, labor, supplies needed). Additional implementation outcomes include acceptability (i.e., degree of satisfaction); feasibility (i.e., practicality of strategy); and sustainability (i.e., maintenance of intervention) [57]. Secondary student-level outcomes will be assessed to understand behavioral impact of the implementation strategy on student weight status, family food security, and dietary behaviors. The EPIS framework will also support evaluation of key implementation determinants and processes over the evaluation period to triangulate implementation and behavioral outcome data.

### Community advisory board

To meaningfully guide this work and ensure our methods are grounded in community member voices, over the last 2 years we have recruited and retained a Community Advisory Board (CAB) comprising individuals (*N* = 10) from academia (*n* = 2), non-profit organizations (*n* = 2), the Philadelphia Department of Public Health (*n* = 1), food service representatives (*n* = 1), former teachers (*n* = 1), parents (*n* = 1), and students in high schools (*n* = 2). This CAB was developed in 2023 at the start of the needs assessment phase (Task 1) and has served as an invaluable sounding board for the 5-year study; for Task 1 (needs assessment) all CAB members reviewed interview guides, analysis protocols, and co-designed dissemination products for our work. All CAB members receive financial compensation for their involvement as acknowledgement of their expertise and effort. Now in its second year, the CAB serves a more embedded role within our research and has supported recruitment of schools, fundraising, partnerships with other organizations in the city, and dissemination efforts beyond peer-reviewed literature. The CAB will continue to support our work and play a greater role related to Task 2–5; examples are outlined in the methods and discussion sections.

### Aim 1: use a community-engaged procedure to develop an equity-focused implementation strategy through a cluster randomized design

**Fig. 2:**
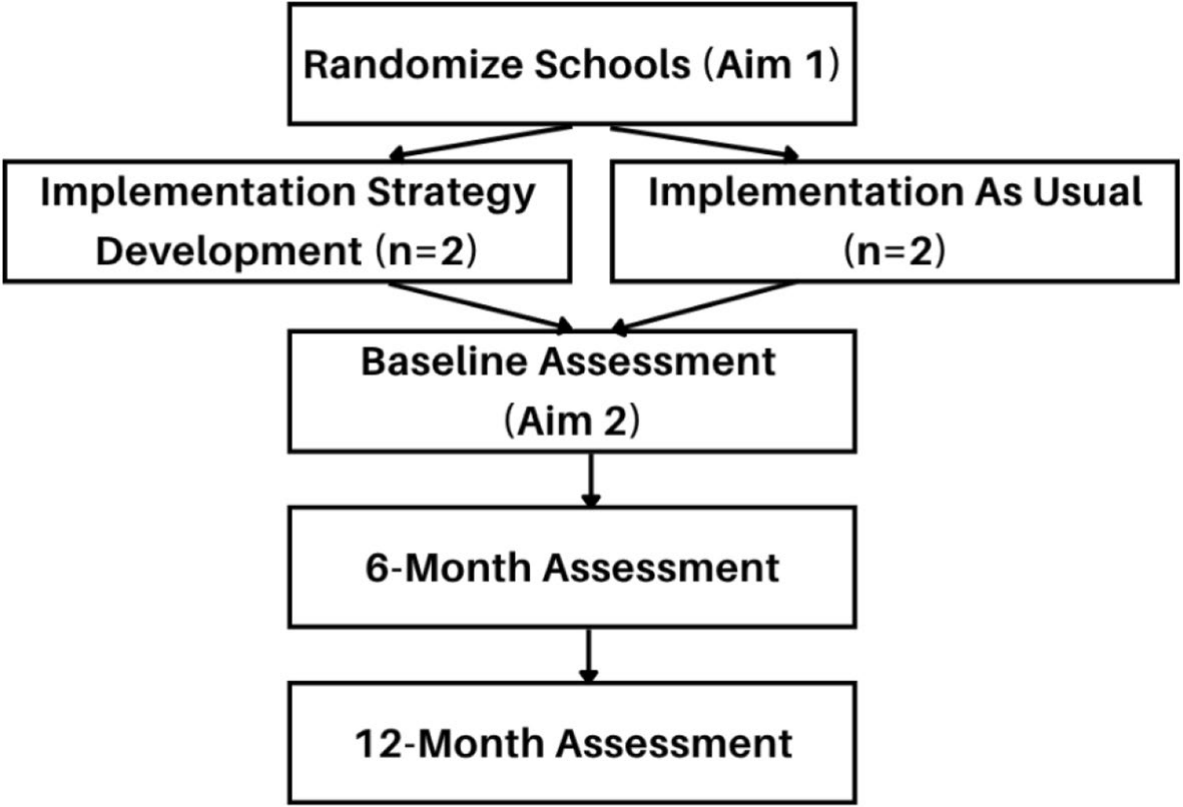
Flow chart of hybrid trial

Informed by findings from the needs assessment (Task 1) [47], this aim will comprise identifying goals for implementation and objectives (Task 2), the development and tailoring of implementation strategies (Task 3), and implementation protocols (Task 4) for USM implementation through a cluster-randomized pilot design. From the schools in the needs assessment sample, two will be block randomized to receive implementation strategy selection support and two will implement USM as usual as a waitlist comparison group, before going through strategy development the following year (Fig. 2). Schools will not be blinded to this randomization given that they will be asked to sign a collaboration agreement and will be told whether they are completing mapping first or assigned to the waitlist condition. Eligible schools are those that have participated in our needs assessment or have agreed to collaboration through a formal agreement and are a public, non-charter school within the SDP.

We will systematically map barriers and strengths identified in Task 1 to USM implementation provided by diverse school and district representatives (i.e., food service providers, teachers, administrators, students, and parents) on to potential strategies based on prior work [58]. The goal of establishing performance objectives (Task 2) will be determined by our findings from Task 1 and by the key goal of increasing student participation in USM. For Task 3, strategies can be from one of many categories including planning (e.g., building stakeholder buy-in); education (e.g., training implementers); financial (e.g., incentives); structural (e.g., create teams); management (e.g., audit and feedback); or policy-related (e.g., licensure standards) [37]. Instead of the initial compilation of strategies developed for clinical settings, which use clinical terms (i.e., patients, providers), [37] our team will primarily consult strategy compilations tailored to the education [58] and community settings [59]. This process ensures we are following the most recent literature and guidance based on pragmatic trials. Grounded in GTE constructs, one potential implementation strategy from the “planning” category could be to assemble a group of students for an advocacy group, who would take a leading role in promoting school meal participation and collaborate with school food service. For Task 4, materials and protocols will be developed grounded with specific attention to the four GTE constructs [7]. These materials will outline what messages, methods, and materials are needed to carry out the chosen strategy through an equity lens. For example, if “planning” is the implementation strategy, specific communication practices may be created to recruit students and engage them in leadership activities based on needs in Task 1 [7].

#### Recruitment and engagement

All schools who participated in Task 1 [47] will be re-engaged and invited to participate in the next phase of the project, with additional schools recruited in collaboration with our CAB and the district to secure a sample of 4 schools. We will ask all schools to sign a collaboration agreement (See additional file 1) that states they agree to be involved in the project to be randomized to the first pilot or the waitlist comparison condition. Once received, we will block randomize schools by pairing the sample (2 in each group) based on characteristics such as geographic location, size, mode of food service (i.e., satellite or full-service kitchen), and student/family demographics.

Grounded in the GTE and EPIS frameworks [7, 53], this implementation mapping aim will be completed with representatives (at least 3) and students from each of the 2 intervention schools. We will meet with pilot school principals early in the process to identify suitable individuals who are invested in the school meals program; necessary individuals to recruit are administrators (i.e., principals) and food service providers given their essential roles in steering meal provision practices. We will aim to recruit at least 1 student, parent, and teacher from each school through distributing flyers (See additional file 2) to schools and targeted sampling through the principal and other administrators to ensure we find individuals who are committed to the process and meetings. We will hold ~ 5 meetings during/after school (based on school team preference) roughly 3–4 weeks apart to accomplish Tasks 2–4 and prepare for full rollout. For all participants, we will distribute informed consent forms (and assent for students) to facilitate data collection and provide a $25 gift card per participant per meeting attended.

#### Data collection procedures and measures

We will utilize predominantly qualitative methods for this strategy development aim through a series of semi-structured meetings with school representatives. Each planning meeting will accomplish part of a mapping task (Tasks 2–4); workbooks will be developed by the research team to distribute to all attendees to facilitate discussion. These workbooks will be developed through review of prior examples of successful toolkits for implementation strategy development [60] in collaboration with our CAB and the school district. Meetings for Task 2 (identify outcomes) will focus on reviewing data from Task 1 (needs assessment), getting feedback from school representatives on findings, and setting goals for implementation. Example prompts will include: “What findings shocked you or stood out?” and “what do you want to see change with the school meals program?” to arrive at clear goals and desired improvements for the program.

Meetings for Task 3 (develop strategies) will focus on taking the desired goals and objectives and developing strategies for improving implementation. We will facilitate refining ideas and asking representatives to collectively rate the difficulty and complexity of chosen strategies (i.e., easy, moderate, hard) and cite potential barriers to implementation. Example prompts for discussion include: “What strategies or practices do you think we could try to address some of these challenges to school meals?” and “What barriers would we need to consider for implementing this strategy?”. Finally for Task 4 (develop protocols) we will collectively finalize the chosen strategies and begin to “backwards plan” for these by discussing what materials/supplies, funding, training, and other support is needed to execute these strategies (prompt: “what do we need to implement this strategy?”) and to decide on the timing, intensity, and frequency of implementing them. Example prompts include: “What funding do we need to get this strategy/project implemented?” and “How often/much are we going to do this [strategy]” for each of the strategies the group selects to test out. We will also discuss who will lead the deployment of each strategy. This will facilitate creation of protocols in alignment with reporting recommendations for implementation strategies [61]. We will record all meetings and have them professionally transcribed verbatim.

#### Analyses

Aligning with the purpose of this aim, we will use a deductive coding process to ensure development of strategies grounded in EPIS and GTE frameworks, followed by inductive coding to generate overarching themes of the strategy development process. We will utilize MAXQDA TeamCloud software [62] to conduct team coding of all meeting transcripts and workbook entries from participants. For deductive analyses, we will create a 3-level coding structure to generate codes for 1) each phase of EPIS (separate codes for barriers and facilitators), 2) each construct (i.e., inner, outer setting, innovation factors, bridging factors), and 3) each quadrant of the GTE with “barrier” and “potential strategy” within each component, similar to prior uses of EPIS for strategy development [63–65]. This will facilitate coding of the transcripts to a code within each level (if applicable) or a code within 1 or 2 levels depending on the data and group decision. For example, during initial mapping meetings a school/district representative may bring up challenges to addressing student stigma because of the limitations with the menu or current meal service format. This kind of example would be coded under “Exploration-Barriers” (EPIS) and under “Reduce Deterrents-Strategy” (GTE) because of the alignment with both our implementation process and health equity frameworks. Analyzing our data in this multifaceted way will hold the research team accountable for spotting equity “blind spots” and for matching pragmatic/logistical challenges with potential strategies in later meetings.

The team will conduct parallel coding of the first 1–2 transcripts to ensure intercoder reliability, followed by consensus coding in pairs to resolve coding disagreements. We chose this process because it facilitates more accurate coding given a relatively small sample size (*n* = 2 schools in first pilot) with large volumes of data for each school (roughly 1.5 h per meeting; ~ 5 meetings total per school yielding ~ 10 transcripts) requiring more in-depth analyses. Use of the GTE framework for implementation strategy development has not been documented before; thus, the more in-depth approach will take longer than using EPIS alone. We will refer to the notes taken in each meeting to support and refine the coding protocol over time.

### Aim 2: evaluate implementation and student-level outcomes of the strategy using a hybrid type III implementation-effectiveness design

Following the hybrid type III approach, [56] a longitudinal convergent (Quantitative–Qualitative) mixed-methods design will investigate the impact of this trial. Primary implementation outcomes of penetration/reach will be calculated through analyzing participation rates from breakfast and lunch service across the implementation period. We will create an implementation costing measure to calculate the financial cost of each implementation strategy. Additional outcomes of acceptability (i.e., are representatives satisfied with this strategy?), feasibility (i.e., was this simple to carry out?), and sustainability (i.e., could this be maintained?) will be assessed from the perspective of implementers. Implementation objectives identified in Task 2 will be evaluated through brief, semi-structured interviews with implementing representatives*.* Secondary student health outcomes of weight status, food insecurity, and dietary behaviors will be analyzed over the 2-year implementation period.

#### Participants and procedures

Intervention school teams will receive coaching during the implementation phase with monthly check-in meetings at school sites or via phone/Zoom if necessary (~ 15–30 min) to support implementation efforts and retention of schools across the intervention period. Implementation leaders and other front-line implementers (i.e., food service staff) from intervention schools will be asked to complete surveys and brief interviews to address implementation outcomes. Administrators and food service providers will be asked to participate in interviews which will facilitate the development of the costing measure. Penetration and behavioral data for all 4 schools will be collected before Aim 1 begins and throughout study completion. Table 1 illustrates the implementation (in green) and behavior/health outcome (in blue) measures we will collect, the source, data collection period, and the timing/frequency of these measures for the hybrid type III implementation-effectiveness study.

**Table 1:** Hybrid type III study: overview of variables, data source, collection period, and timing/frequency of data collection

	**Variable**	**Source**	**Data Collection Period**	**# Time Points/Frequency**
**Implementation Outcomes**	Penetration (Meal Participation)	Division of Food Services	Daily for Breakfast and Lunch	Monthly Average (%) per student
	Cost	Cost Survey and Interview	Fall and spring each year	4 (2 in each trial year)
	Acceptability	Implementation Survey	Fall and spring in first year	2
	Feasibility	Implementation Survey	Fall and spring in first year	2
	Sustainability	Implementation Survey	Fall and spring each year	4 (2 in each trial year)
	Implementation Determinants	Check-in Meetings	Monthly	18 (9 each year)
**Behavior/Health Outcomes**	Race, ethnicity, economic disadvantage	District database	Fall of each year	2
	Weight Status/BMI	Office of Health Services	Fall of each year	2
	Student hunger, self-reported participation, dietary behavior	Office of Research and Evaluation	Spring of each year (student survey)	2
	Attendance	District Database	Daily for each student	Monthly Average (%) per student

#### Data collection and primary outcomes

The number of breakfast and lunch meals served (linked to student ID) will provide data on penetration, facilitating assessment of equitable access to low-income students. For cost of implementation, Our team will develop a bottom-up costing strategy and measure the costs for each implementation strategy that results from the mapping process, following guidance from experts in economic evaluation of implementation [49, 66] and in collaboration with our CAB and school district partners. We will do this by establishing categories of resource use for each strategy (e.g., labor, materials, travel) and developing initial measures to capture time and effort of all implementing participants at the school sites. We will then conduct a budget impact analysis and costconsequence analysis to enhance understanding of the value for each strategy and determine potential for scale-up. Validated surveys adapted for the school context by the principal investigator (see additional file 3) will provide data on acceptability, feasibility, and sustainability of the implementation strategy by each member of the implementation team [67–69]. Finally, brief (i.e., 20–30 min) interviews will be held with key implementing representatives (identified in Task 1) to identify the degree to which implementation objectives were met grounded in the GTE toward the end of the first year of implementation [38, 44, 45]. We will also ask about overarching facilitators and barriers during these interviews, mapping determinants to the Implementation and Sustainment phases of EPIS. To support replicability of our protocol, all surveys, interview guides, coding materials, and other pertinent documents will be made publicly available on our lab website and through article publication.

#### Data sources and secondary outcomes

Besides the student survey (to report hunger/dietary intake) which is administered each spring, all data points are gathered by school or district-level staff. Demographic data are gathered each year during registration; data will be monitored for change over time but only baseline data will be analyzed. Identified Student Percentage is created to show the percent of students in each school whose families are receiving federal assistance such as Supplemental Nutrition Assistance Program, Women, Infants, and Children program, or other programs. These data are then housed at the district to provide a binary variable of “economic disadvantage” based on participation (or non-participation) in a federal assistance program. At each school, nurses are trained to collect height and weight data each year as part of student health screening to provide BMI percentiles. Meal participation is gathered every day as students enter their unique ID number into the point-of-sale system when taking a meal; these data are uploaded to the district database. Survey data are stored in the districts’ Office of Research and Accountability. Finally, attendance is gathered at each school and daily records are uploaded to the district site.

Several large blinded (i.e., name and other identifying information removed) datasets housed at the SDP will be merged including the Division of Food Service; student body mass index (BMI) data from the Office of Student Health Services; race, ethnicity, economic disadvantage, and attendance from the District Performance Office; and food insecurity/survey data from the Office of Research and Accountability. Due to the PI’s ongoing training activity and collaboration with the district, they are in full support of sharing these data to support research activity. Student BMI data are collected by school nurses in the spring of each year to assess weight status, providing a continuous variable. Household food insecurity will be assessed via the US Department of Agriculture 6-item survey (additional file 4), [70, 71] and dietary behavior from the student survey to examine frequency of consuming various food groups such as fruits, vegetables, processed/fried foods, sugar-sweetened vegetables, and other items (see additional file 5).

#### Analyses

Overall, the longitudinal data collection at baseline, 6 months, and 12 months following the inception of the trial will facilitate assessment of change over time and identification of factors that influence the outcomes. For the implementation outcomes, penetration will be analyzed by calculating the proportional change in number of low-income students participating in USM from prior to enacting the implementation strategy at baseline, 6 months, and 12 months following the start of the trial. For the costing measure, labor costs will be iteratively analyzed through the bottom-up approach to collect time and effort data from all implementing individuals; salary data will be obtained for all SDP positions through review of publicly available records. Receipts and invoices for all materials and miscellaneous costs will be analyzed. All costs will be calculated specific to each strategy to allow for accurate reporting and budget impact assessment showing net financial costs resulting from the strategies. The costconsequence analysis will be conducted to show the comparison of the costs and outcomes of each strategy in comparison to the waitlist condition (i.e., treatment as usual) [49, 72]. Data from the acceptability, feasibility, and sustainability surveys will be processed and aggregated at the school level to provide overall means. Interview data on other outcomes identified through the mapping procedure will be analyzed deductively through the GTE and Proctor outcomes framework [7, 57] followed by mapping ongoing determinants to the EPIS framework in the Implementation and Sustainment phases, providing tangible barriers and facilitators across the trial period [53]. This will enhance continuation of determinants analysis from Aim 1 and support adaptation/tailoring of the implementation strategies over time in response to needs/challenges at each school. Such adaptation is recommended by implementation science experts as a means to enhance equity through collaborations with community and clinical partners [43].

For the behavior/health outcomes, we will analyze data at baseline and 12 months to examine changes over time given that BMI, household food insecurity, and dietary behavior data are collected annually. Prior to analysis data will be cleaned following guidance from experts [73] and will entail checking for errors in the data such as incorrect, missing values, inconsistencies, and duplicates. We will work with the SDP Office of Research and Accountability to develop a cleaning plan and follow their best practices for data management including data security to maintain confidentiality. Datasets for each behavioral outcome will be created, to provide a school- and student-level dataset in R Software (Vienna, Austria). Exploratory hierarchical multiple regression models will be employed for each of the behavioral outcomes using students as the unit of observation and schools as the unit of analysis. Differences between intervention and control conditions will be determined, and school-specific effects, i.e., random effect for schools will be included in our models. This will allow correlations between students within schools which are nested within the intervention condition. We will also run regression models to examine change over time and what variables are associated with this change, examining trajectories among the intervention and comparison group. This will allow calculation of Intraclass Correlation Coefficient, providing necessary pilot data [74, 75] for a subsequent high-powered clustered trial.

The research team is well-equipped to support the monitoring of participants and schools in this study. We will take all precautions to address these discomforts, including training study staff to be aware of these potential issues and comfortable interacting with school personnel, parents, and adolescents. If participants seem uncomfortable or reactive to the study measures, they will have the option to discontinue data collection. Safety reports will also be created and sent to Temple University’s Data Safety and Monitoring Board (DSMB). Results of all assessments will be reviewed with the support of the mentorship team to determine whether subjects have experienced any adverse events. Any serious events, even though unlikely, will be reported within 24 h to Temple’s Institutional Review Board, as appropriate. If any protocol modifications occur, we will update the trial registration immediately.

### Qualitative rigor

Below we document steps that will be taken to demonstrate rigor in our qualitative analyses [76, 77].

### Validity/credibility

The research team will develop a coding consensus document and logbook, which will become “living documents” that guide decision-making and alignment with qualitative coding. We will take several steps to achieve intercoder reliability. For Aims 1 and 2 initial agreement will be calculated by each team member coding the meeting transcripts and aiming for 75% agreement or above on coded constructs. Following this, one team member will code all remaining transcripts, and another team member will act as secondary coder; they will independently code transcripts and conduct consensus coding to modify documents and discuss coding interpretations, resolving any disagreements. We will integrate observation data triangulate the interview data, especially where coders had areas of uncertainty or disagreement.

### Reliability/dependability

The team will keep an audit trail called a “coding decisions protocol” in which they will log all changes made to coding throughout the consensus approach. After each meeting in Aim 1 we will disseminate meeting notes and main takeaways to school implementation teams through a shared Google drive to ensure they can make changes and add additional notes if desired, enhancing the collaborative process. For interviews for Aim 2 we will send transcripts to implementation team members so they can review their responses and send clarifications and updates. Finally, to enhance our interpretation of the findings we will regularly debrief with CAB members who give input on coding and analysis procedures, holding us accountable to confront our subjectivity and potential bias in coding.

### Confirmability

Finally, to address confirmability, we will take extensive field notes from interviews and school observations across both study aims (and after virtual interviews if applicable). We will continue reflective practice in team meetings, using discussions to adapt coding definitions and inclusion criteria based on new data that challenged our positionality.

### Project timeline

The anticipated timeline for this project is shown in Table 2. We plan to complete this trial over a 3-year period which will take until the end of the project funding period (February 2028). Adaptations may occur which will be tracked and documented throughout the trial.

**Table 2.**
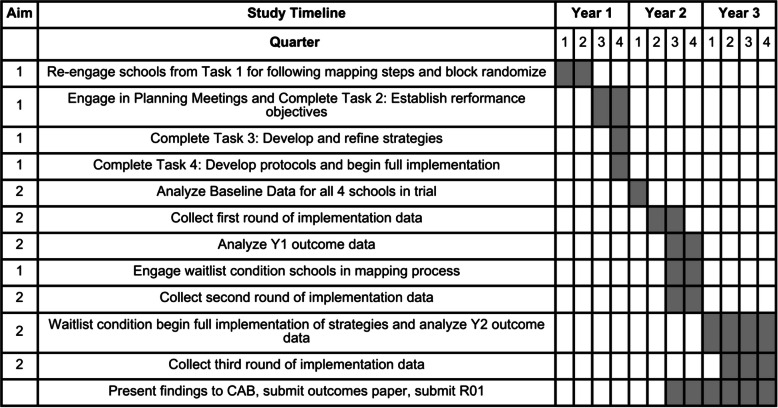
Project timeline

## Discussion

This study will contribute to the fields of implementation science and health policy in multiple ways. First, we highlight the intersection of implementation science and health equity principles to address food insecurity and obesity prevention [43]. Many studies in school settings fail to consider organizational (i.e., school context), external (i.e., policy, systems, community), and financial (i.e., costs of implementation) factors that can be profound barriers to real-world impact and equitable implementation [78, 79]. “To our knowledge, this is one of the first randomized trials to integrate implementation mapping, equity frameworks, and cost analysis for the largest food security safety net for school-aged children living in the US. This project will directly address this gap by identifying implementation strategies through a community-engaged approach [80]. Implementation scientists have stressed the need for more concrete health equity frameworks; [39–41, 81] thus, this study will directly contribute to advancements in the field by integrating the revised CFIR (V2), [44, 45] the EPIS framework, and the GTE [7]. Collectively, these frameworks provide a more comprehensive lens (e.g., CFIR for inner/outer context, EPIS for phase-based guidance, GTE for equity alignment) and support a rigorous evaluation approach of implementation mapping.

Second, we are developing a community-engaged implementation mapping process. Although implementation mapping is inherently stakeholder-driven, [38, 82] the lack of engagement of students, parents, and other key innovation recipients in prior work [83–85] warrants their involvement and input given the increased focus shared decision making in implementation science [41, 43, 86]. Finally, we are conducting a mixed methods cost analysis of school PSE implementation strategies. Implementation science is embracing cost analysis as a key focus, [46, 48, 49, 87] given the inherent challenges of conducting implementation interventions without considering financial implications [46]. The focus on cost as a key implementation outcome will improve our ability to enhance feasibility and sustainability of PSE interventions for food insecurity and obesity prevention.

Dissemination of this work beyond traditional outlets (i.e., peer-reviewed publications, conference presentations) is a core part of our work and, in collaboration with our CAB, we have engaged in the Designing for Dissemination and Sustainability process [88–90] (paper in press). We actively engage CAB members in the analysis and interpretation of data to proactively plan dissemination products such as website pages, social media posts, infographics, reports, and research briefs, with the goal of transcending beyond traditional audiences for our work. We will continue to meet monthly with SDP partners and share ongoing data collection and analysis updates. Finally, we will engage with other organizations and research teams across the city, nation, and internationally to share lessons learned and project updates, building capacity for equity-focused implementation mapping.

There are multiple challenges for this work to take place. Schools serving low-income populations in the US face myriad challenges to education delivery including low budgets, difficulty retaining teachers and leaders, and trying to meet the needs of a population experiencing high levels of community trauma [91–94]. Although data highlight that USM improves food insecurity, attendance, and by extension academic achievement, the low levels of reimbursement received from the government mean that funding is already low for USM implementation and known barriers such as stigma to participation persist [95]. As such, engaging with schools to conduct research and run a cluster-randomized trial poses logistical challenges but also may unintentionally spotlight “failings” of this program and create distrust among school representatives toward our research team, mirrored in prior literature [86, 96, 97]. Thus, engaging the district from the beginning and partnering with the schools will necessitate that the research team approaches this work with the highest level of respect for those implementing USM and receiving the provision. The strengths of the research team, including experience working with marginalized populations and as licensed teachers in low-income schools, will be leveraged throughout the implementation mapping process. The involvement of our CAB from the beginning is an added strength as they will support engagement practices to ensure shared power and autonomy of our schools throughout this process.

## Summary

This project will accomplish the development and testing of equity-focused implementation strategies for USM to promote improved uptake of healthy school meals among school students in Philadelphia. This intervention will utilize an evidence-based process [38] and test primary implementation outcomes of penetration and cost, [48, 49] and secondary impact on student health outcomes. This research is significant because it will yield a community-engaged implementation mapping procedure to enhance the effectiveness of school-based obesity prevention approaches by increasing equitable access to healthy meals. This implementation mapping process will yield equity-driven strategies which can be successfully implemented in school settings to improve uptake of USM and reduce obesity-related disparities in children and adolescents. Findings may inform national policies on USM and serve as a replicable model for other school districts seeking to implement equity-oriented strategies. Finally, this study outlines a rigorous agenda that holds potential for improving the equity of food insecurity and obesity prevention PSE interventions by identifying, developing, and testing effective implementation strategies to meet the needs of vulnerable children in school-based settings in the US with promise for global application.

## Supplementary Information

Additional file 1. School Meals Collaboration Letter.

Additional file 2. School Meals Recruitment Flyer.

Additional file 3. Implementation Outcome Measures.

Additional file 4. 2024-2025 Food Security Survey.

Additional file 5. Student survey questions.

Additional file 6. Ethics Approval.

Additional file 7. Funding Statement.

Additional file 8. SPIRIT Checklist.

## Data Availability

Data associated with this manuscript can be requested from the corresponding author.

## Abbreviations

BMI: Body Mass Index,
CAB: Community Advisory Board
CFIR: Consolidated Framework for Implementation Research
EPIS: Exploration, Preparation, Implementation, Sustainment framework
GTE: Getting to Equity in Obesity Prevention Framework
NSLBP: National School Lunch and Breakfast Program
SDP: School District of Philadelphia
PSE: Policy, Systems, and Environmental
USM: Universal School meals
