# Supplementary material for: Using implementation mapping to optimize the impact of Universal School meals: a type III hybrid implementation-effectiveness study protocol
Source: Implement Sci Commun. 2025 Oct 1;6:97. doi: 10.1186/s43058-025-00769-y (PMC12486583; doi:10.1186/s43058-025-00769-y)
Supplement: Supplementary file 2 — Additional file 2. School Meals Recruitment Flyer. [file 43058_2025_769_MOESM2_ESM.pdf]

WE NEED YOU!

Are you interested in representing your school community? Are you passionate about making change in your school?

JOIN US!

Your school is taking part in the collaborative between the School District of Philadelphia and Temple University to improve student participation in school meals and we need you! We are looking for **students**, **parents**, **teachers**, **food service staff, climate staff**, and **administration** who are interested in representing their school in this collaboration and providing feedback to the school meals program at you school.

- 3 after-school meetings
- Create strategies to improve student participation in school meals.
- Collaborate with RISE team on strategies and provide feedback

Receive compensation of $25 for your participation in each meeting

Temple University College of Public Health

THE SCHOOL DISTRICT OF PHILADELPHIA

LEARN MORE ABOUT US AT
www.temple.edu/riselab/
or scan the QR Code to visit our website

RISE. RESEARCH IN IMPLEMENTATION SCIENCE TO ENHANCE EQUITY
