# Supplementary material for: Using implementation mapping to optimize the impact of Universal School meals: a type III hybrid implementation-effectiveness study protocol
Source: Implement Sci Commun. 2025 Oct 1;6:97. doi: 10.1186/s43058-025-00769-y (PMC12486583; doi:10.1186/s43058-025-00769-y)
Supplement: Supplementary file 3 — Additional file 3. Implementation Outcome Measures. [file 43058_2025_769_MOESM3_ESM.docx]

**Additional File 3 Implementation Outcome Measures**

| **Outcome** | **Lay definition** | **Implementer-Facing Question (i.e., teacher, food service, admin)** | **Recipient (i.e., student/caregiver) facing question** |
| --- | --- | --- | --- |
| Acceptability | **Acceptability of the Implementation Strategy(ies)**  What, if anything, do you like about the implementation strategy? Is it a good fit for your school? | 1. The implementation strategy(ies) are appealing to me 2. The welcome use of these strategy(ies) 3. I like the school meals implementation strategy(ies) for our school 4. I believe the implementation strategy(ies) are working for our school | 1. The implementation strategy(ies) are appealing to me 2. The welcome use of these strategy(ies) 3. I like the school meals implementation strategy(ies) for our school 4. I believe the implementation strategy(ies) are working for our school |
| Feasibility | **Feasibility of the Implementation Strategy(ies)** How easily do you think these strategies are to implement? | 1. It is/was difficult for me to learn the components of the implementation strategy(ies) 2. It is/was difficult to implement the implementation strategy(ies) while meeting the needs of our student population 3. Delivering the implementation strategy(ies) requires substantial changes to how we operate school meals 4. The goals of the implementation strategy(ies) are realistic for our school |  |
| Reach/Penetration | **Reach/Penetration of the Implementation Strategy(ies)** What is the proportion of students who participate in school meals relative to the school population? | 1. Do you perceive differences in which students participate in (policy provision)? Yes/no 2. [if yes]: What characteristic(s) make students less likely to participate in (policy provision) (select all that apply)  - Minoritized racial or ethnic group - Low socio-economic status - Primary language other than English - LGBTQ+ or minoritized gender - Minoritized religious affiliation - Minoritized cultural identity - Children from single-parent households - Immigrant population - Other (please describe) |  |
| Sustainability | **Sustainability of the Implementation Strategy(ies)** How likely do you think your school is to maintain efforts to advance health equity through school meals? | 1. My school system has enough staff to implement this strategy 2. My school system conducts periodic needs assessments of the community to make sure that the strategy continues to meet their needs 3. My school system is planning for evaluation of the strategy 4. My school system uses evaluation data to monitor and improve the strategy 5. My school system shares the evaluation findings from the strategy to members of the community 6. Leadership within my school encourages the use of evidence-based interventions to guide school meal implementation efforts. 7. My direct supervisor expects me to include research evidence in decision making related to planning the implementation of strategy 8. Evidence-based programs are readily adopted within my school | 1. What barriers or challenges make it harder to access school meals? 2. How would you want to see these challenges fixed or addressed? |
