# Supplementary material for: Using implementation mapping to optimize the impact of Universal School meals: a type III hybrid implementation-effectiveness study protocol
Source: Implement Sci Commun. 2025 Oct 1;6:97. doi: 10.1186/s43058-025-00769-y (PMC12486583; doi:10.1186/s43058-025-00769-y)
Supplement: Supplementary file 4 — Additional file 4. 2024-2025 Food Security Survey. [file 43058_2025_769_MOESM4_ESM.pdf]

THE SCHOOL DISTRICT OF PHILADELPHIA
PHILLY SCHOOL EXPERIENCE SURVEY

2024-25 Philly School Experience Survey for Parents & Guardians

Health and Nutrition

These questions help the District know which schools can use more programs like food pantries and healthy food stands. Please remember, you can skip any questions. This survey is anonymous and individual responses will never be linked to your family or shared with schools or other organizations.

10. In the past 12 months, how often were the following statements true about your household?

	Never	Sometimes	Often	Don't know/Prefer not to answer
The food that I/we bought just didn't last and I/we didn't have money to get more.	○	○	○	○
I/we couldn't afford to eat balanced meals.	○	○	○	○

11. In the past 12 months...

	Yes	No	Don't know/Prefer not to answer
did you ever eat less than you felt you should because there wasn't enough money for food?	◯	◯	◯
were you ever hungry but didn't eat because there wasn't enough money for food?	◯	◯	◯

12. In the past 12 months, did you or other adults in your household ever cut the size of your meals or skip meals because there wasn't enough money for food?

- ◯ No
- ◯ Yes, only 1 or 2 months
- ◯ Yes, some months but not every month
- ◯ Yes, almost every month
- ◯ Don't know/prefer not to answer

69%

Back Next
