# Supplementary figures and images for: Using implementation mapping to optimize the impact of Universal School meals: a type III hybrid implementation-effectiveness study protocol
Source: Implement Sci Commun. 2025 Oct 1;6:97. doi: 10.1186/s43058-025-00769-y (PMC12486583; doi:10.1186/s43058-025-00769-y)

Additional File 4: Student Survey Items


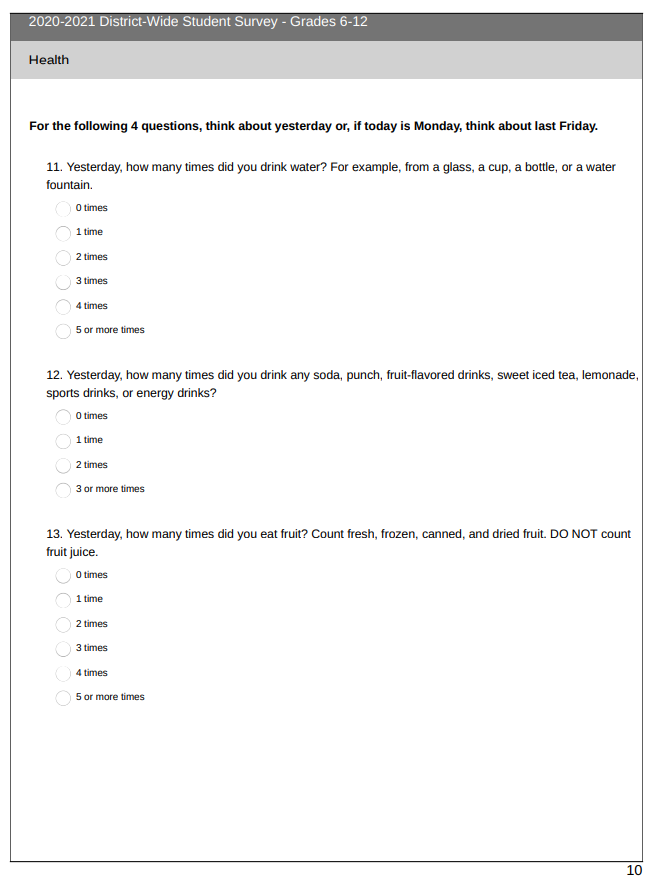


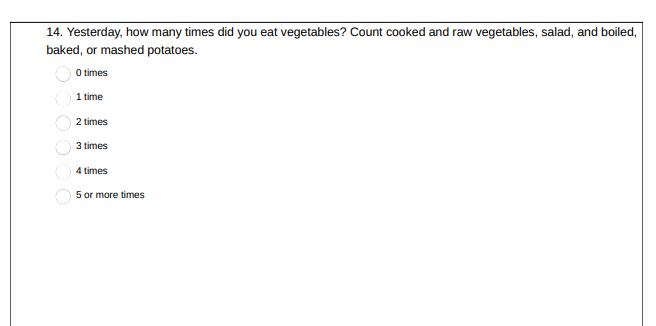


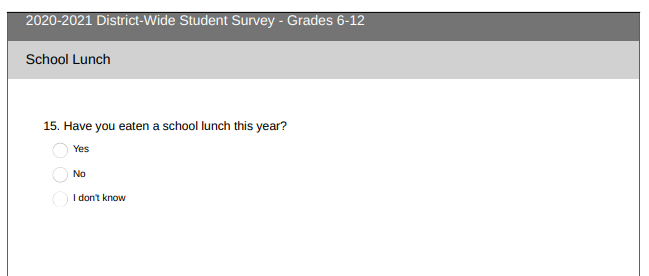


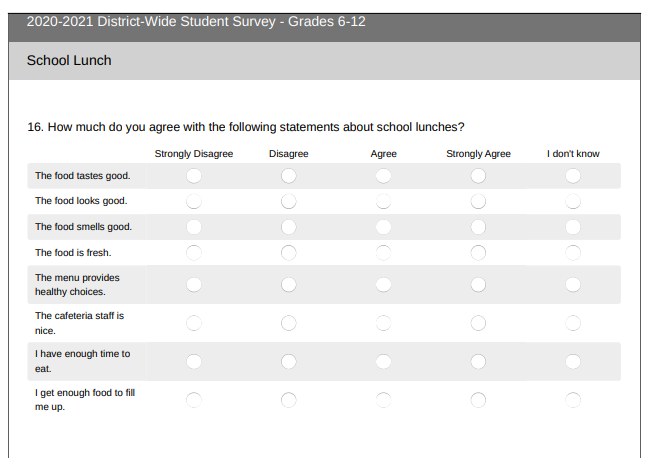


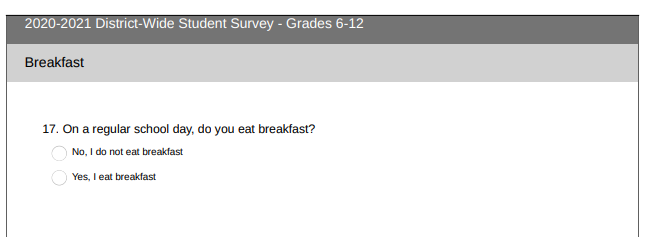

Supplement: Supplementary file 5 — Additional file 5. Student survey questions. [file 43058_2025_769_MOESM5_ESM.docx]
