# Supplementary material for: Using implementation mapping to optimize the impact of Universal School meals: a type III hybrid implementation-effectiveness study protocol
Source: Implement Sci Commun. 2025 Oct 1;6:97. doi: 10.1186/s43058-025-00769-y (PMC12486583; doi:10.1186/s43058-025-00769-y)
Supplement: Supplementary file 6 — Additional file 6. Ethics Approval. [file 43058_2025_769_MOESM6_ESM.pdf]

Temple University
Office of the Vice President for Research

Research Integrity & Compliance
Student Faculty Center
3340 N. Broad Street, Suite 427
Philadelphia PA 19140

Institutional Review Board
Phone: (215) 707-3390
Fax: (215) 204-4609
e-mail: irb@temple.edu

Amendment Approval

Date: 12-Aug-2024

Protocol Number:	29859
PI:	GABRIELLA MCLOUGHLIN
Review Date:	12-Aug-2024
Committee:	A1
Risk:	Minimal risk
Sponsor:	NATIONAL HEART, LUNG, AND BLOOD INSTITUTE/NIH/DHHS
Project Title:	Using implementation mapping to maximize equity of Universal School Meals.

On 12-Aug-2024, the IRB approved the amendments requested in Submission # **29859-0015**. A summary of the approved Amendments is below:

1- Uploading the study details (consent forms, recruitment emails, and measurement tools) for Aims 2 and 3 of the study. This includes updating the protocol as well.

*If you amended the consent form for a non-Exempt study, you can access your IRB-approved, stamped consent document or consent script through ERA. Open the "Attachments" tab within the approved submission (# **29859-0015**) and open the stamped documents by clicking the View link next to each document. The stamped documents are labeled as such. Copies of the IRB approved stamped consent document or consent script must be used in obtaining consent.*

Please contact the IRB at (215) 707-3390 if you have any questions.

If you would like to tell us how we are doing, please complete this 5-minute Satisfaction Survey: https://forms.gle/9EcgYGDEEANnvMw37
