# Supplementary material for: Using implementation mapping to optimize the impact of Universal School meals: a type III hybrid implementation-effectiveness study protocol
Source: Implement Sci Commun. 2025 Oct 1;6:97. doi: 10.1186/s43058-025-00769-y (PMC12486583; doi:10.1186/s43058-025-00769-y)
Supplement: Supplementary file 7 — Additional file 7. Funding Statement. [file 43058_2025_769_MOESM7_ESM.pdf]

Department of Health and Human Services
National Institutes of Health
NATIONAL HEART, LUNG, AND BLOOD INSTITUTE

Notice of Award
FAIN# K01HL166957
Federal Award Date
01/31/2024

Recipient Information

1. Recipient Name
TEMPLE UNIVERSITY-OF THE COMMONWEALTH SYSTEM OF HIGHER EDUCATION
1801 N BROAD ST
PHILADELPHIA, PA 19122

2. Congressional District of Recipient
02

3. Payment System Identifier (ID)
1231365971A1

4. Employer Identification Number (EIN)
231365971

5. Data Universal Numbering System (DUNS)
057123192

6. Recipient's Unique Entity Identifier
QD4MGHFDJKU1

7. Project Director or Principal Investigator
Gabriella Mcloughlin, PHD

gabriella.mcloughlin@temple.edu
732-397-4905

8. Authorized Official
Jeffery Tolliver
jeff.tolliver@temple.edu
215-204-6875

Federal Agency Information

9. Awarding Agency Contact Information
Jasmine Johnson
Grant Management Specialist
NATIONAL HEART, LUNG, AND BLOOD INSTITUTE
jasmine.johnson@nih.gov
(301) 827-8177

10. Program Official Contact Information
Sharon M Smith
Program Officer
NATIONAL HEART, LUNG, AND BLOOD INSTITUTE
smithsh1@mail.nih.gov
3014350053

Federal Award Information

11. Award Number
5K01HL166957-02

12. Unique Federal Award Identification Number (FAIN)
K01HL166957

13. Statutory Authority
42 USC 241 42 CFR 52

14. Federal Award Project Title
Using implementation mapping to maximize equity of school-based policies for obesity prevention

15. Assistance Listing Number
93.837

16. Assistance Listing Program Title
Cardiovascular Diseases Research

17. Award Action Type
Non-Competing Continuation

18. Is the Award R&D?
Yes

Summary Federal Award Financial Information

**19. Budget Period Start Date** 02/01/2024 **– End Date** 01/31/2025	
**20. Total Amount of Federal Funds Obligated by this Action**	$171,504
20 a. Direct Cost Amount	$158,800
20 b. Indirect Cost Amount	$12,704
**21.** Authorized Carryover	
**22.** Offset	
**23.** Total Amount of Federal Funds Obligated this budget period	$171,504
**24. Total Approved Cost Sharing or Matching, where applicable**	$0
**25. Total Federal and Non-Federal Approved this Budget Period**	$171,504
**26. Project Period Start Date** 02/15/2023 **– End Date** 01/31/2028	
**27.** Total Amount of the Federal Award including Approved Cost Sharing or Matching this Project Period	$343,008

28. Authorized Treatment of Program Income
Additional Costs

29. Grants Management Officer - Signature
Tammi SIMPSON

30. Remarks
Acceptance of this award, including the "Terms and Conditions," is acknowledged by the recipient when funds are drawn down or otherwise requested from the grant payment system.

SECTION I – AWARD DATA – 5K01HL166957-02

Principal Investigator(s):
Gabriella Mcloughlin, PHD

Award e-mailed to: nih@temple.edu

Dear Authorized Official:

The National Institutes of Health hereby awards a grant in the amount of $171,504 (see "Award Calculation" in Section I and "Terms and Conditions" in Section III) to TEMPLE UNIV OF THE COMMONWEALTH in support of the above referenced project. This award is pursuant to the authority of 42 USC 241 42 CFR 52 and is subject to the requirements of this statute and regulation and of other referenced, incorporated or attached terms and conditions.

Acceptance of this award, including the "Terms and Conditions," is acknowledged by the recipient when funds are drawn down or otherwise requested from the grant payment system.

Each publication, press release, or other document about research supported by an NIH award must include an acknowledgment of NIH award support and a disclaimer such as "Research reported in this publication was supported by the National Heart, Lung, And Blood Institute of the National Institutes of Health under Award Number K01HL166957. The content is solely the responsibility of the authors and does not necessarily represent the official views of the National Institutes of Health." Prior to issuing a press release concerning the outcome of this research, please notify the NIH awarding IC in advance to allow for coordination.

Award recipients must promote objectivity in research by establishing standards that provide a reasonable expectation that the design, conduct and reporting of research funded under NIH awards will be free from bias resulting from an Investigator's Financial Conflict of Interest (FCOI), in accordance with the 2011 revised regulation at 42 CFR Part 50 Subpart F. The Institution shall submit all FCOI reports to the NIH through the eRA Commons FCOI Module. The regulation does not apply to Phase I Small Business Innovative Research (SBIR) and Small Business Technology Transfer (STTR) awards. Consult the NIH website http://grants.nih.gov/grants/policy/coi/ for a link to the regulation and additional important information.

If you have any questions about this award, please direct questions to the Federal Agency contacts.

Sincerely yours,

Tammi SIMPSON
Grants Management Officer
NATIONAL HEART, LUNG, AND BLOOD INSTITUTE

Additional information follows

Cumulative Award Calculations for this Budget Period (U.S. Dollars)

**Salaries and Wages**	$100,000
**Fringe Benefits**	$28,800
**Personnel Costs (Subtotal)**	$128,800
**Other**	$30,000
**Federal Direct Costs**	$158,800
**Federal F&A Costs**	$12,704
**Approved Budget**	$171,504
**Total Amount of Federal Funds Authorized (Federal Share)**	$171,504
**TOTAL FEDERAL AWARD AMOUNT**	$171,504
**AMOUNT OF THIS ACTION (FEDERAL SHARE)**	$171,504

**SUMMARY TOTALS FOR ALL YEARS** (for this Document Number)		
**YR**	**THIS AWARD**	**CUMULATIVE TOTALS**
2	$171,504	$171,504
3	$170,477	$170,477
4	$170,477	$170,477
5	$170,477	$170,477

Recommended future year total cost support, subject to the availability of funds and satisfactory progress of the project

Fiscal Information:

Payment System Identifier: 1231365971A1
Document Number: KHL166957A
PMS Account Type: P (Subaccount)
Fiscal Year: 2024

IC	CAN	2024	2025	2026	2027
HL	8475183	$171,504	$170,477	$170,477	$170,477

Recommended future year total cost support, subject to the availability of funds and satisfactory progress of the project

NIH Administrative Data:
PCC: HHEF N / **OC**: 41035 / **Released**: SIMPSON, Tammi 01/30/2024
Award Processed: 01/31/2024 09:38:20 AM

SECTION II – PAYMENT/HOTLINE INFORMATION – 5K01HL166957-02

For payment and HHS Office of Inspector General Hotline information, see the NIH Home Page at http://grants.nih.gov/grants/policy/awardconditions.htm

SECTION III – STANDARD TERMS AND CONDITIONS – 5K01HL166957-02

This award is based on the application submitted to, and as approved by, NIH on the above-titled project and is subject to the terms and conditions incorporated either directly or by reference in the following:

a. The grant program legislation and program regulation cited in this Notice of Award.
b. Conditions on activities and expenditure of funds in other statutory requirements, such as

Version: 13 - 8/3/2022 12:59 PM | Generated on: 1/31/2024 9:38 AM

those included in appropriations acts.

c. 45 CFR Part 75.
d. National Policy Requirements and all other requirements described in the NIH Grants Policy Statement, including addenda in effect as of the beginning date of the budget period.
e. Federal Award Performance Goals: As required by the periodic report in the RPPR or in the final progress report when applicable.
f. This award notice, INCLUDING THE TERMS AND CONDITIONS CITED BELOW.

(See NIH Home Page at http://grants.nih.gov/grants/policy/awardconditions.htm for certain references cited above.)

Research and Development (R&D): All awards issued by the National Institutes of Health (NIH) meet the definition of "Research and Development" at 45 CFR Part§ 75.2**.** As such, auditees should identify NIH awards as part of the R&D cluster on the Schedule of Expenditures of Federal Awards (SEFA). The auditor should test NIH awards for compliance as instructed in Part V, Clusters of Programs. NIH recognizes that some awards may have another classification for purposes of indirect costs. The auditor is not required to report the disconnect (i.e., the award is classified as R&D for Federal Audit Requirement purposes but non-research for indirect cost rate purposes), unless the auditee is charging indirect costs at a rate other than the rate(s) specified in the award document(s).

This institution is a signatory to the Federal Demonstration Partnership (FDP) Phase VII Agreement which requires active institutional participation in new or ongoing FDP demonstrations and pilots.

An unobligated balance may be carried over into the next budget period without Grants Management Officer prior approval.

This grant is subject to Streamlined Noncompeting Award Procedures (SNAP).

This award is subject to the requirements of 2 CFR Part 25 for institutions to obtain a unique entity identifier (UEI) and maintain an active registration in the System for Award Management (SAM). Should a consortium/subaward be issued under this award, a UEI requirement must be included. See http://grants.nih.gov/grants/policy/awardconditions.htm for the full NIH award term implementing this requirement and other additional information.

This award has been assigned the Federal Award Identification Number (FAIN) K01HL166957. Recipients must document the assigned FAIN on each consortium/subaward issued under this award.

Based on the project period start date of this project, this award is likely subject to the Transparency Act subaward and executive compensation reporting requirement of 2 CFR Part 170. There are conditions that may exclude this award; see http://grants.nih.gov/grants/policy/awardconditions.htm for additional award applicability information.

In accordance with P.L. 110-161, compliance with the NIH Public Access Policy is now mandatory. For more information, see NOT-OD-08-033 and the Public Access website: http://publicaccess.nih.gov/.

This award provides support for one or more clinical trials. By law (Title VIII, Section 801 of Public Law 110-85), the "responsible party" must register "applicable clinical trials" on the ClinicalTrials.gov Protocol Registration System Information Website. NIH encourages registration of all trials whether required under the law or not. For more information, see http://grants.nih.gov/ClinicalTrials_fdaaa/

Recipients must administer the project in compliance with federal civil rights laws that prohibit discrimination on the basis of race, color, national origin, disability, age, and comply with applicable conscience protections. The recipient will comply with applicable laws that prohibit discrimination on the basis of sex, which includes discrimination on the basis of gender identity, sexual orientation, and pregnancy. Compliance with these laws requires taking reasonable steps to provide meaningful access to

Version: 13 - 8/3/2022 12:59 PM | Generated on: 1/31/2024 9:38 AM

persons with limited English proficiency and providing programs that are accessible to and usable by persons with disabilities. The HHS Office for Civil Rights provides guidance on complying with civil rights laws enforced by HHS. See https://www.hhs.gov/civil-rights/for-providers/provider-obligations/index.html and https://www.hhs.gov/.

- Recipients of FFA must ensure that their programs are accessible to persons with limited English proficiency. For guidance on meeting the legal obligation to take reasonable steps to ensure meaningful access to programs or activities by limited English proficient individuals, see https://www.hhs.gov/civil-rights/for-individuals/special-topics/limited-english-proficiency/fact-sheet-guidance/index.html and https://www.lep.gov.
- For information on an institution's specific legal obligations for serving qualified individuals with disabilities, including providing program access, reasonable modifications, and to provide effective communication, see http://www.hhs.gov/ocr/civilrights/understanding/disability/index.html.
- HHS funded health and education programs must be administered in an environment free of sexual harassment; see https://www.hhs.gov/civil-rights/for-individuals/sex-discrimination/index.html. For information about NIH's commitment to supporting a safe and respectful work environment, who to contact with questions or concerns, and what NIH's expectations are for institutions and the individuals supported on NIH-funded awards, please see https://grants.nih.gov/grants/policy/harassment.htm.
- For guidance on administering programs in compliance with applicable federal religious nondiscrimination laws and applicable federal conscience protection and associated anti-discrimination laws, see https://www.hhs.gov/conscience/conscience-protections/index.html and https://www.hhs.gov/conscience/religious-freedom/index.html.

In accordance with the regulatory requirements provided at 45 CFR 75.113 and Appendix XII to 45 CFR Part 75, recipients that have currently active Federal grants, cooperative agreements, and procurement contracts with cumulative total value greater than $10,000,000 must report and maintain information in the System for Award Management (SAM) about civil, criminal, and administrative proceedings in connection with the award or performance of a Federal award that reached final disposition within the most recent five-year period. The recipient must also make semiannual disclosures regarding such proceedings. Proceedings information will be made publicly available in the designated integrity and performance system (currently the Federal Awardee Performance and Integrity Information System (FAPIIS)). Full reporting requirements and procedures are found in Appendix XII to 45 CFR Part 75. This term does not apply to NIH fellowships.

Treatment of Program Income:
Additional Costs

SECTION IV – HL SPECIFIC AWARD CONDITIONS – 5K01HL166957-02

Clinical Trial Indicator: Yes
This award supports one or more NIH-defined Clinical Trials. See the NIH Grants Policy Statement Section 1.2 for NIH definition of Clinical Trial.

NHLBI FUNDING GUIDELINES

NIH is currently operating under a Continuing Resolution (See NIH Guide Notice NOT-OD-24-007) and this award is being issued in accordance with the NHLBI FY 2024 Operating Guidelines which can be found at: https://www.nhlbi.nih.gov/research/funding/general/current-operating-guidelines

MENTORED CAREER DEVELOPMENT AWARD

Mentored CDA recipients are required to devote a minimum commitment equivalent of 9 calendar person months (75% of their full-time appointment at the applicant institution) to the career development and research objectives of the program specified in FOA, the policy and additional details are found here:

https://grants.nih.gov/grants/policy/nihgps/HTML5/section_12/12.3.6_level_of_effort.htm. T he recipient may supplement the NHLBI salary contribution with non-federal funds up to a level that is consistent with the institution's salary scale, the policy, including additional details and exceptions are found here:
https://grants.nih.gov/grants/policy/nihgps/HTML5/section_12/12.8.1_salaries_and_fringe_benefits.htm

MENTOR'S REPORT
Please note that a concise statement from the awardee's mentor must be included in the RPPR. The statement should address progress and performance, see Section 7.1 of the NIH RPPR Instruction Guide.

NHLBI ADJUSTMENTS FOR SALARY BASED AWARDS
Salary funds provided on NHLBI research grants will be adjusted if investigators receive careertype salary based awards. Examples of such awards include the Independent Scientist Award (K02, formerly K04) and other similar awards as described in the November 1993 Circulation article (Vol. 88, No. 5, Part 1). In the event that such an award is made for an investigator receiving salary support from an NHLBI grant, the Institute must be informed in writing within 30 days from the start date of the award so that any required adjustment can be made.

NON-COMPETING RENEWAL (SNAP)
The NIH requires the use of the Research Performance Progress Report (RPPR) for all Type 5 progress reports. The RPPR and other documents applicable to this SNAP grant are due the 15th of the month preceding the month in which the budget period ends (e.g., if the budget period ends 11/30, the due date is 10/15). Please see http://grants.nih.gov/grants/rppr/index.htm for additional information on the RPPR.

PRIOR APPROVAL REQUEST
It is recommended that applicable prior approval requests be submitted via the eRA Commons Prior Approval Module (link: prior_approval (nih.gov)). Please refer to Part II Chapter 8 of the NIH Grants Policy Statement for the activities and/or expenditures that require NIH approval at http://grants.nih.gov/grants/policy/nihgps/nihgps.pdf

SPREADSHEET SUMMARY
AWARD NUMBER: 5K01HL166957-02

INSTITUTION: TEMPLE UNIV OF THE COMMONWEALTH

Budget	Year 2	Year 3	Year 4	Year 5
Salaries and Wages	$100,000	$100,000	$100,000	$100,000
Fringe Benefits	$28,800	$28,800	$28,800	$28,800
Personnel Costs (Subtotal)	$128,800	$128,800	$128,800	$128,800
Other	$30,000	$30,000	$30,000	$30,000
TOTAL FEDERAL DC	$158,800	$158,800	$158,800	$158,800
TOTAL FEDERAL F&A	$12,704	$11,677	$11,677	$11,677
TOTAL COST	$171,504	$170,477	$170,477	$170,477

Facilities and Administrative Costs	Year 2	Year 3	Year 4	Year 5
F&A Cost Rate 1	8%	8%	8%	8%
F&A Cost Base 1	$158,800	$145,965	$145,965	$145,965
F&A Costs 1	$12,704	$11,677	$11,677	$11,677

Version: 13 - 8/3/2022 12:59 PM | Generated on: 1/31/2024 9:38 AM
