# Supplementary material for: Using implementation mapping to optimize the impact of Universal School meals: a type III hybrid implementation-effectiveness study protocol
Source: Implement Sci Commun. 2025 Oct 1;6:97. doi: 10.1186/s43058-025-00769-y (PMC12486583; doi:10.1186/s43058-025-00769-y)
Supplement: Supplementary file 1 — Additional file 1. School Meals Collaboration Letter. [file 43058_2025_769_MOESM1_ESM.docx]

Dr. Gabriella M. McLoughlin
1300 Cecil B. Moore Ave.
Ritter Annex, 9^th^ floor
Philadelphia, PA 19122

Dear Dr. McLoughlin,

_______ School wishes to participate in the Philadelphia School Meals collaboration being conducted by you and your team in the College of Public Health at Temple University. We understand that all schools agreeing to participate will take part in a lottery to determine when they will receive the intervention. The possible semesters for the start of official intervention planning are: Spring 2025 (to test in Fall 2025) or Fall 2025 (to test in Spring 2026).

Meetings for this project will begin in Spring 2025 to build capacity and recruit representatives for the planning period, regardless of when we will implement the intervention. I understand that this multi-year program is designed to improve school meal participation and overall program delivery, and in semesters when the program is not being conducted in our school, data collection will still occur. The anticipated project end date is June 2027; this is so that we can understand how the program can be sustained over time.

As part of our participation, we agree to:

- Recruit representatives from our school who are parents, food service providers, students, classroom teachers, climate staff, custodial/building engineers, community liaisons, and other school community members to be a part of the planning process;
- Recruit and select students to be leaders and get involved with the project (as needed);
- Meet with the RISE team regularly in the planning and testing phase at a time that works for our school representatives;
- Grant permission for the RISE team to be present in the school setting for training, data collection, and during planning meetings;
- Assist in obtaining consent from all participants, especially students and parental assent;
- Encourage staff to complete surveys during the intervention period (who will be financially compensated);
- Provide short periods of time for the administration of student surveys and interviews during class time (only once per year for students; twice per year for staff/faculty) between 2025-2027;
- Designate a contact person in food services to work with our staff and communicate updates to the rest of the team;
- Support data request efforts made to the district so that we have all data necessary to carry out the project evaluation. This includes factors such as attendance, academic achievement, student survey data, and other data as applicable;
- Meet with your staff on a regular basis as needed;

**In return we expect the RISE Team (Led by Gabriella McLoughlin) to:**

- Engage with our school representatives on a regular basis, ideally once per month, to learn how school meals are being implemented and provide feedback;
- Consult with us on strategies for selecting representatives that will engage in implementation mapping;
- Depending on what strategies our representatives create, provide training to school staff on how these will be implemented;
- Support implementation of the strategy and engage with the Division of Food Services and Eat Right Philly to ensure resources are allocated to your school;
- Provide financial compensation for the primary representatives at our school for participating in meetings ($25 per meeting) and for completing surveys and interviews as part of data collection;
- Be present, as requested, at 1-2 (more, if applicable) important school events per year such as staff trainings and professional development to answer questions on the project and maintain presence within the school;
- Seek funding to support implementation from outside agencies and foundations, and support grant applications;
- Communicate the findings of the program in our school as such data becomes available and to collaborate on additional presentations of data as needed;
- Prepare a report for us describing the results of the project as it relates to our school when the project is completed;
- Train personnel in our District to be able to continue the program beyond the end of the grant;
- Meet with our staff on a regular basis as needed;
- Provide advice (as needed, based on skills) to help us design and implement other programs;
- Assist us in gaining access to other University programs and activities; and
- Connect us with community organizations who can support the intervention.
- Other (please specify): _________________________________________________

We understand that all costs associated with the project, other than those mentioned above, will be covered by the grant.

We look forward to a productive collaborative relationship on this project and expect that the benefits to our students and staff will be considerable.

Sincerely,

[Signed and printed name of administrator]
